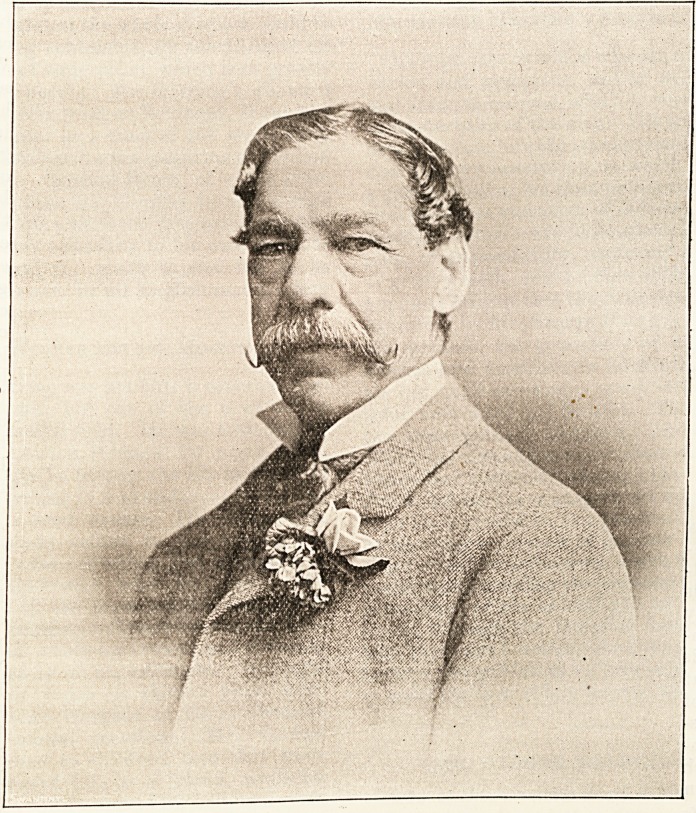# A Great Benefactor to Our Hospitals

**Published:** 1906-11-10

**Authors:** 


### HOSPITAL ADMINISTRATION.

### CONSTRUCTION AND ECONOMICS.

### A GREAT BENEFACTOR TO OUR HOSPITALS.

THE LATE MR. GEORGE HERRING.

It is with great regret and personal sorrow, that we have to announce the sudden death of Mr. George Herring, one of the most practical benefactors and friends of the hospitals, the nineteenth century has supplied. Mr. George Herring was in his seventy-fourth year, and so recently as the 25th ultimo he attended a meeting of the Distribution Committee of the Hospital Sunday Fund, where he displayed all his accustomed energy and common sense, in the conduct of the business before the meeting. Later in the same week he was shooting on his estate Putteridge Park, near Luton. On Sunday the 28th ultimo he was taken ill, was found to be suffering from appendicitis, was operated upon by Mr. Lockwood of St. Bartholomew's Hospital on Tuesday, and did well after the operation until Friday morning, when he became suddenly worse and died from heart failure. His sudden illness of course prevented him from handing to the Fund the balance still due of some £275 but he had already paid £11,000, being 25 per cent. of the amount raised by the Hospital Sunday Fund in September. Mr. Herring's final contribution did not fall due until the end of the Sunday Fund's financial year—*i.e.* on October 31—when it was possible to ascertain exactly the whole sum due on the basis of his offer for the current year. We feel confident that Mr. George Herring's keen business character makes it certain, that before his death, arrangements were made to fulfil every obligation he had entered into. We expect, it will be found, that he has provided for the payment of the cheque in question and for the future development and encouragement of the Metropolitan Hospital Sunday Fund.

**Personal Characteristics.**

As a man Mr. George Herring was shrewd, businesslike, honourable, intelligent, clear sighted, kind hearted and generous to a fault. The writer knew him well, and in the course of some years' intercourse and knowledge of his character Mr. Herring was never known to say an unkind thing about anybody, or to offer criticism of any kind except that which was justified by the facts, or calculated to prove helpful to the movement, or individual, to which or to whom it related. He was a most entertaining, cheerful, happy companion, and it was one of the delights of his life, to spend several weeks of each year at Monte Carlo, where he was seldom or never seen at the tables, but where he enjoyed the beauty of the gardens, the brightness of the sunshine, and the easy, careless life, for which this pleasure resort is famous, all the world over.

Mr. Herring was one of the most modest and contented of men. He held, that honours conferred upon a man for generosity displayed towards charities, or objects of public utility, were dishonouring, rather than the reverse, and for his part, he neither desired nor would he accept any such recognition, because, it seemed to him, that to do so, might tend to diminish the good, which he desired should follow, every attempt he made, to leave the world a little happier and better, than he found it. Always reticent, he never talked about himself, his family, or his experiences. He always refused, to sit for his portrait, to give away his photographs, or to name the photographer from whom they could be obtained. This feature of his character was based upon the feeling, that the personal journalism of the present day is opposed to the best interests of individuals, and calculated to weaken, rather than to strengthen, character, in men and women. So he became, from the very modesty and retiring nature of the man, a person of continuous interest to pressmen, and to all who have to do with the movements and work of prominent personages.

### His Keen Interest in Hospitals.

Mr. George Herring had a very keen interest in the welfare of hospitals supported by voluntary contributions. His view was, that the tendency of the day, in hospital affairs, inclined too much in the direction of prodigal expenditure upon buildings, some of which could be well dispensed with, and most of which were conceived, on so lavish a scale, as to represent, when completed, a grievous waste of public money. No doubt these views had much to do with his selection of the Metropolitan Hospital Sunday Fund, as the chief vehicle for his gifts to hospitals. The methods of the Hospital Sunday Fund afforded great pleasure to Mr. Herring, from the circumstance, that all the money entrusted to it, is entirely devoted to hospital maintenance, and none of it is ever given for building purposes. As we pointed out, two years ago, there is, however, another side to this question of hospital rebuilding, which we know he was fully alive to, from conversations we had with Mr. Herring. The metropolitan hospitals, and indeed most of the older hospitals throughout the country, during the last fifteen years, have had to face a problem, which necessarily presents itself, at least once in every hundred years,—namely, the necessity for reconstruction or rebuilding. It is the fact, that every sick house or hospital must be rebuilt, periodically, and the managers have no alternative but to face this contingency, and as far as practicable to provide for it, in advance, by accumulating invested funds. In the past our forbears and sires found the money to build the hospitals originally, and to reconstruct and rebuild them as occasion required. We, of the present generation, have been passing through the greatest period of hospital rebuilding, which the country has ever experienced, and it is greatly to the credit of all concerned, that at least fifteen million pounds have been raised, or contributed by the public to, British hospitals for this purpose, during the last fifteen years. The greater part of this rebuilding has now been carried out and paid for. The voluntary hospitals and their supporters may therefore look forward, to a period of rest, after prodigal liberality, provided every supporter of a hospital will show the business acumen of Mr. George Herring, by selecting the institutions which most appeal to him, and then continue to give systematically, to them, year by year.

### The Salvation Army Scheme and the King's Fund.

Altogether Mr. George Herring has contributed a sum of nearly £90,000 to the hospitals through the Hospital Sunday Fund. Amongst other gifts mention must be made of the £100,000 placed in the hands of the Salvation Army to be employed, first in settling poor people on neglected land in the United Kingdom, in establishing them as petty cultivators, and supporting them and their families, until the land becomes productive. The money, sunk in this way, is to be gradually paid back by the settlers, and then to be handed by the Salvation Army to King Edward's Hospital Fund, in twenty-five annual payments, of £4,000 each. Mr. Herring objected to emigration, because he held, that as England contains millions of acres uncultivated, or partly cultivated, we ought first to provide every man, who needs employment and is willing to work, with occupation, upon these uncultivated lands, rather than permit him to go abroad as an emigrant. England expends fifty millions every year in the purchase of produce, almost the whole of which could be raised, Mr. Herring contended, by substituting his plan for the plan of emigration, which is so greatly favoured by many people. Mr. Herring's scheme is now being tentatively tried at Boxstead, about two miles from Colchester on a tract of 360 acres, upon which suitable buildings for the reception of the men have been erected. General Booth, of whose practical business qualities Mr. Herring formed a high opinion, has been left a perfectly free hand, to carry out the experiments according to his own methods and principles, and the result cannot fail to be watched, with interest, by all Englishmen of every class.

### An Active Worker for the Hospitals.

Mr. George Herring was not only a generous contributor to hospitals, but one of the most active workers in the hospital field. He was treasurer of the North West London Hospital for many years, and in that capacity he became the life and soul of the institution, devoting to it abundance of time and energy, as well as much money. His death must prove a most serious matter to this institution, which, under his administration, has increased in efficiency and importance year by year. Mr. Herring was one of the most regular and active members of the Distribution Committee of the Hospital Sunday Fund, where his counsel and criticism were greatly valued by all his colleagues. Amongst other hospitals for which he showed great sympathy, the Children's Hospital in Great Ormond Street deserves special mention. Mr. George Herring was devoted to children, and they have never failed to regard him as a friend, while he could never resist a child's appeal for help and sympathy.

### His Coadjutors and Practical Philanthropy.

Mr. Herring first publicly exhibited his sympathy for hospitals, as the almoner of the late Baron Hirsch, who entrusted to him the distribution of all his winnings on the turf. We have reason to think, that Mr. Herring's close association in business with another generous philanthropist of the highest intelligence, Mr. H. L. Bischoffsheim, had much to do with the direction of his sympathies towards hospitals, and to certain enterprises, which from their character, secured the wise use of money, to help workers, in various fields of industry, whose means were as small, as their opportunity for advancement. So Mr. Herring, in conjunction with Mr. Howard Morley, brought his business knowledge to bear upon the building and management of a club for ladies, earning their own livelihood, which he made self-supporting on business lines. He further developed the same idea by instituting other similar enterprises. Had he lived, there is little doubt, that the Herring system of clubs for women workers, might have rivalled the Rowton Houses, in popularity and success. He also took a great personal interest in a Haven for old people, which he established at Maidenhead, near his own beautiful home, on the banks of the river Thames.

### Mr. George Herring's Example and Memorials.

Of his private charities who can speak? They were, varied and numerous, and so were the generosity and kindheartedness, which formed two of the chief characteristics of the man. His method of personal investigation, into the management and administration of every institution, to which he contemplated a gift, as well as his close personal attention, to the circumstances and needs of individual cases, offer an example, which every philanthropist, who has a due sense of responsibility, conferred by the possession of great wealth, might follow with advantage. We hope and believe, that the quiet strength of character exhibited by Mr. George Herring, the excellence of his methods, the generosity of his gifts, and the wisdom and unselfishness he displayed, throughout his life, may encourage, many rich people, to consider their ways and be wise. The indebtedness of the metropolitan hospitals, through the money which has actually come to them directly, from his hands, and indirectly, through the Hospital Sunday Fund, afford the managers an opportunity to exhibit their gratitude, in a practical and useful form, by securing a portrait of the late Mr. George Herring for their board-rooms, which might bear a brief inscription, recording the practical wisdom and great generosity, of his annual gift to the London hospitals, through the Hospital Sunday Fund. We hope that each hospital, which may adopt some such form of recognition as we here suggest, will not fail to make the fact widely known, through the Press.

---

### MR. GEORGE HERRING'S WILL.

Owing to the directions found in the will, a Memorial Service, not the funeral as at first intended, was held at Brookwood Cemetery on Wednesday. The bequests prove how self-contained and purposeful a mind Mr. Herring had, for despite the great personal influence to which he was open in favour of particular hospitals, he has given, practically, all his money to the two great funds.

The following are the main features of the late Mr. George Herring's will, after providing annuities for his wife and brother-in-law, and leaving a number of pecuniary and other legacies to his brother, friends and servants, £5,000 is bequeathed to the North-West London Hospital, of which testator was treasurer; £500 to the Maidenhead Cottage Hospital;

£1,000 to the Society for Charitable Relief; and £5,000 to the Salvation Army Social Fund.

The will provides for the selection by certain friends, in the order named in the will, of articles of art or vertu from among the effects at testator's house at Hamilton Place. Subject to these gifts, No, 1 Hamilton Place and its contents are to be sold, and the proceeds paid over "to King Edward's Hospital Fund for London as a slight acknowledgment of his Majesty's most gracious message to me, and in the confident hope that having regard to the ultimate object of this gift good prices will be realised at the sale."

The whole of the residue, which is expected to exceed half a million, is to be applied by the trustees for the benefit and purposes of the Metropolitan Hospital Sunday Fund.

There are two codicils made just before Mr. Herring's death which provide for the due carrying out of his gift of £100,000 to the Salvation Army Land Scheme, and for further legacies.

Provision is also made for a further endowment of "The Haven of Rest" Almshouses, Maidenhead, of which Mr. Herring was the founder.

Mr. Herring leaves instructions that his body should be cremated, and that the urn containing his ashes should be deposited under the sundial at his "Haven of Rest" Maidenhead.

## Figures and Tables

**Figure f1:**